# Kisspeptin modulates gamma-aminobutyric acid levels in the human brain

**DOI:** 10.1016/j.psyneuen.2021.105244

**Published:** 2021-07

**Authors:** Alexander N. Comninos, Lisa Yang, James O’Callaghan, Edouard G. Mills, Matthew B. Wall, Lysia Demetriou, Victoria C. Wing, Layla Thurston, Bryn M. Owen, Ali Abbara, Eugenii A. Rabiner, Waljit S. Dhillo

**Affiliations:** a Division of Diabetes, Endocrinology & Metabolism, Imperial College London, UK; b Department of Endocrinology, Imperial College Healthcare NHS Trust, London, UK; c Invicro, London, UK; d Nuffield Department of Women’s and Reproductive Health, University of Oxford, UK

**Keywords:** Kisspeptin, Gamma-aminobutyric acid (GABA), Sexual behaviour, Reproductive behaviour, Limbic system, Anterior cingulate cortex

## Abstract

Gamma-aminobutyric acid (GABA) is a key inhibitory neurotransmitter that has been implicated in the aetiology of common mood and behavioural disorders. By employing proton magnetic resonance spectroscopy in man, we demonstrate that administration of the reproductive neuropeptide, kisspeptin, robustly decreases GABA levels in the limbic system of the human brain; specifically the anterior cingulate cortex (ACC). This finding defines a novel kisspeptin-activated GABA pathway in man, and provides important mechanistic insights into the mood and behaviour-altering effects of kisspeptin seen in rodents and humans. In addition, this work has therapeutic implications as it identifies GABA-signalling as a potential target for the escalating development of kisspeptin-based therapies for common reproductive disorders of body and mind.

## Highlights

• The neuropeptide, kisspeptin, is a crucial regulator of reproductive behaviour.
• The molecular mechanisms that govern its behavioural action in humans are unknown.
• Kisspeptin or vehicle was administered to 19 healthy men.
• Anterior cingulate cortex gamma-aminobutyric acid (GABA) levels were assessed.
• Kisspeptin decreased GABA, a key inhibitory peptide, in this major limbic region.

## 1 Introduction

The neuropeptide kisspeptin sits at the apex of the reproductive axis, acting as the master regulator of downstream reproductive hormone release (Babwah, 2020), with an evolutionary history spanning over 540 million years (Wang et al., 2020). Emerging data demonstrates that kisspeptin also modulates related mood and sexual behaviours (Comninos et al., 2017, Comninos et al., 2018, Yang et al., 2020b), through its extensive distribution in the human limbic system (Muir et al., 2001).

Pre-clinical animal models suggest that kisspeptin achieves these effects in part through modulation of the key inhibitory neurotransmitter gamma-aminobutyric acid (GABA) (Adekunbi et al., 2017, Defazio et al., 2014, Di Giorgio et al., 2014, Li et al., 2015, Neal-Perry et al., 2009). However, crucially there is currently no data in this regard to substantiate the applicability of these findings in humans. Indeed, up to now there have been no studies of kisspeptin’s interactions with neuropeptides or neurotransmitters of interest outside the classical reproductive axis in humans. Thus, we sought to provide the first evidence of whether an in vivo change in neurotransmitter levels following kisspeptin administration could be demonstrated in humans, and specifically whether a change in central GABA levels could be detected during kisspeptin administration in healthy men. To achieve this, we employed magnetic resonance spectroscopy (MRS) as a robust noninvasive imaging technique that can determine total endogenous GABA concentrations in the human brain (Mullins et al., 2014).

These data would provide a conceptual advance and fundamental mechanistic data for the neurophysiological actions of kisspeptin, as well as the escalating development of kisspeptin-based therapies for common reproductive disorders of body and mind (Comninos et al., 2017, Yang et al., 2020b, Yang and Dhillo, 2016).

## 2 Materials and methods

### 2.1 Study design

The study was approved by the regional ethics committee (Riverside Research Ethics Committee, London, United Kingdom, REC ref: 17/LO/1504) and was performed in accordance with the Declaration of Helsinki. Informed consent was obtained after participants were given the opportunity to review the study information and ask questions. A hormonal within-participant intervention study assessing GABA changes in the ACC had not previously been carried out when we designed this study. However, based on the previous literature of sample sizes required to identify a functionally significant change of 10–15% in GABA signal, we required a dataset from 11 to 19 participants (Bollmann et al., 2015, Northoff et al., 2007). Therefore, to allow for dropouts and exclusions, we recruited 27 participants.

Participants attended two study visits, one for kisspeptin and one for vehicle, in random order at least 7 days apart. Participants were blinded to the identity of the infusions. The cross-over design, in which the participants acted as their own control, minimised variability and enhanced power. All study visits commenced in the morning to control for circadian hormonal changes. Baseline psychometric questionnaires were completed prior to the scan to assess for abnormal anxiety, reward and sexual desire; traits which may confound the analyses, with all scores within normal limits (Table 1).

**Table 1:** Participant baseline characteristics.

		Mean ± SEM
Age (years)		26.5 ± 1.1
BMI (kg/m^2^)		23.9 ± 0.4
Baseline Reproductive Hormones	LH (IU/L)	2.6 ± 0.2
	Testosterone (nmol/L)	20.4 ± 0.9
Baseline trait scores		
STAI-Y Trait		36.6 ± 1.8
BIS		19.4 ± 0.8
BAS	Drive	11.6 ± 0.4
	Fun	12.2 ± 0.5
	Reward	17.5 ± 0.4
SDI	Dyadic	44.7 ± 1.4
	Solitary	14.1 ± 1.4
	Total Score	62.9 ± 2.1

Notes: BMI = Body Mass Index; LH = Luteinizing Hormone; STAI-Y Trait = State-Trait Anxiety Inventory to assess trait anxiety; BIS = Behavioural Inhibition System Scale to assess sensitivity to punishment anticipation; BAS = Behavioural Activation System Scale to assess sensitivity to desired goals, fun and reward. SDI = Sexual Desire Inventory to assess dyadic and solitary sexual desire. Results confirmed normal baseline hormone levels, no active anxiety trait, reward responsiveness or sexual desire abnormalities that could confound MRS analysis. n = 19.

**Fig. 1:**
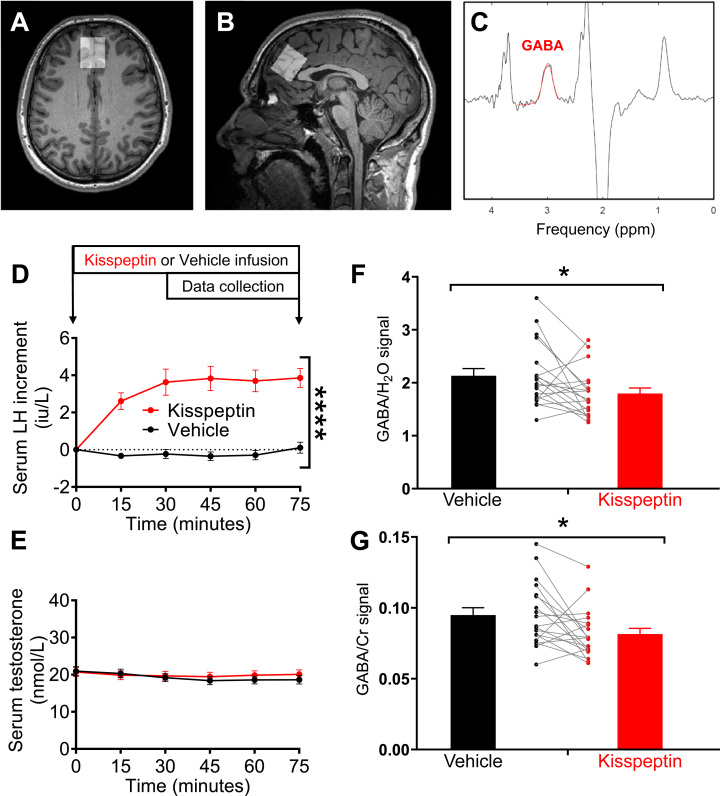
Effects of kisspeptin (1 nmol/kg/h) on reproductive hormones and GABA levels in the anterior cingulate cortex. (A) Representative voxel placement in the ACC coronal and (B) sagittal section. (C) Representative MRS spectrum (black) and GABA peak fit (red). (D) Kisspeptin increased circulating LH but not (E) testosterone levels compared to vehicle (mean ± SEM, ****p < 0.0001, mixed model analysis of variance, n = 19). (F) Kisspeptin decreased GABA levels corrected to water (F), (t18 = 2.173, p = 0.043) and (G) creatine (Cr) (t18 = 2.443, p = 0.025) (mean ± SEM and within-participant raw data, paired *t*-test, n = 19) (For interpretation of the references to colour in this figure legend, the reader is referred to the web version of this article).

On arrival, participants were asked to remove any metal on their person and change into loose hospital scrubs. After a period of acclimatisation, two intravenous cannulae (one in each arm) were inserted (one for blood sampling and the second for infusion of 1nmol/kg/h kisspeptin-54 or vehicle) with timings, sampling and data collection as per Fig. 1. The kisspeptin dose and protocol timings were selected to ensure steady-state levels of kisspeptin during the data collection period (Comninos et al., 2017), while avoiding downstream testosterone increases which would occur later following kisspeptin exposure (Jayasena et al., 2011). MRS was initiated at 30 min from the start of infusion to allow circulating kisspeptin levels to reach steady state as per our previous work (Comninos et al., 2017).

### 2.2 MRI/MRS acquisition

Participants were imaged at 3 T with a Siemens Trio scanner (Siemens Healthcare) using a 32-channel head coil. To aid accurate placement of the MRS voxel, a high resolution T1-weighted structural image was acquired using a 3D Sagittal MPRAGE sequence with parameters: TR/TE = 2300 ms/2.98 ms, FOV = 256mmx240mm, voxel size = 1 × 1 × 1 mm^3^, 160 slices. For GABA-edited MRS, a volume of interest (25 × 25 × 30 mm^3^) was placed on the ACC at the brain midline (Fig. 1A and B). A MEGA-PRESS sequence was used for GABA detection at 3 ppm. Details of the MRS acquisition parameters are provided in the Supplementary Material.

### 2.3 GABA quantification

Fitting and quantification of GABA signal (Fig. 1C and Supplementary Material Fig. S1) was performed in MATLAB (R2016a, MathWorks) using Gannet software (GABA‐MRS Analysis Tool v2.0, http://www.gabamrs.com/downloads)(Edden et al., 2014). To ensure sufficient quality, a threshold of 12% fitting error (provided by Gannet) was used to reject spectra of poor fit as previously published (Edden et al., 2014). Where a single fit did not meet this criterion, both visits were excluded to allow paired analysis of 19 datasets from an original set of 27. GABA signals are reported relative to unsuppressed water and creatine (Cr) signals with corrections as previously described (Mullins et al., 2014). Values for GABA and water relaxation constants at 3 T, as well as sequence efficiency and macromolecular correction terms, were as previously published (Harris et al., 2017, Mullins et al., 2014). The GABA/Cr was calculated as a ratio of fitted signal integrals produced by Gannet (Harris et al., 2017). Signals from macromolecular populations were not suppressed or removed and therefore contributed to the GABA estimates (often referred to as GABA+).

## 3 Results and discussion

We measured total endogenous GABA levels in the human brain, using MRS, during kisspeptin compared to vehicle administration in 19 healthy men (mean ± SEM: age 26.1 ± 1.2 y; BMI 23.6 ± 0.6 kg/m^2^; Table 1). Participants served as their own control to ensure intra-participant validity of the data. We selected the anterior cingulate cortex (ACC), a key limbic structure, as our region of interest based on its central role in kisspeptin behavioural physiology, evidenced by its dense kisspeptin receptor distribution (Muir et al., 2001) and its markedly enhanced activity following kisspeptin administration in men exposed to sexual and couple-bonding stimuli (Comninos et al., 2017). Furthermore, GABA levels particularly in the ACC have been studied extensively using MRS methodologies, and have been shown to be reliable both within and between neuroimaging sessions, thereby ensuring the robustness of our findings (Northoff et al., 2007).

As expected, an intravenous infusion of kisspeptin (1 nmol/kg/h) increased circulating luteinizing hormone (LH) to similar levels, as previously described using this protocol (Comninos et al., 2017, Comninos et al., 2018), confirming that this dose of kisspeptin was biologically active (Fig. 1D). Furthermore, by performing MRS shortly after initiation of the kisspeptin infusion, we ensured that there was no increase in downstream testosterone at the time of MRS assessment (Fig. 1E), in order to assign the neurochemical changes detected to be due to kisspeptin alone.

Having established the biological activity of the kisspeptin infusion, we assessed GABA levels in the ACC during kisspeptin compared to vehicle administration. We and others have previously demonstrated that kisspeptin can cross the blood-brain-barrier to exert central effects (Comninos et al., 2017, d’Anglemont de Tassigny et al., 2017). In this study, we observed a significant decrease in total endogenous GABA levels in the ACC during kisspeptin compared to vehicle administration (14.1–15.7%, Fig. 1F and G). This potent inhibitory effect of kisspeptin on GABA levels endured when corrected both against water (GABA/H_2_O: 14.1% reduction, t(18) = 2.17, p = 0.043) and creatine (GABA/Cr: 15.7% reduction, t(18) = 2.44, p = 0.025)(Fig. 1F and G). Crucially, a similar magnitude of GABA change to that we observed in our study, has been previously been reported in psychological studies with functional significance (Bollmann et al., 2015, Cleve et al., 2015), including in modulating impulsivity (Silveri et al., 2013). Therefore, we demonstrate that kisspeptin can substantially decrease central levels of the key inhibitory neurotransmitter GABA in the human brain, providing a putative mechanism for kisspeptins actions in humans. Given that GABA disturbances may underly several mood and behavioural disorders (Silveri et al., 2013, Bollmann et al., 2015), further studies in patients with these disorders may provide a fruitful avenue for further clinical research.

Consequently, we provide the first evidence in humans that corroborates data in rodent models suggesting that kisspeptins effects are in part mediated through bidirectional interactions with GABA in the limbic system (Di Giorgio et al., 2014, Li et al., 2015, Adekunbi et al., 2017;Comninos, 2016) itself an established brain system for mood and behaviour. Taken together, our data that kisspeptin can decrease central GABA, provides a new understanding of the mechanism of kisspeptin’s established actions to ultimately stimulate ACC and other limbic brain activity in response to sexual and couple-bonding stimuli in humans (Comninos et al., 2017, Comninos et al., 2018).

Although kisspeptin may be directly acting on kisspeptin receptors known to be in the ACC (Muir et al., 2001) to decrease GABA, it is also important to note that additional mechanisms may be involved. Indeed, as well as increasing downstream reproductive hormones which can themselves have several behavioural roles (Riordan et al., 2018), kisspeptin can also modulate serotonin, dopamine, vasopressin, glutamate and nitric oxide signalling (reviewed in Mills et al., 2018). Thus, the effects we see in the current and previous behavioural studies may be the result of direct actions of kisspeptin on its receptor as well as interactions with these additional pathways, with kisspeptin serving as the overall conductor.

In this and our previous work demonstrating behavioural effects due to an identical kisspeptin administration protocol (Comninos et al., 2018, Comninos et al., 2017, Yang et al., 2020b, Yang et al., 2020a), we have employed peripheral administration as it is not possible to administer kisspeptin directly into the brain in humans, and we acknowledge that this is different from physiological kisspeptin release. However, the peripheral kisspeptin levels achieved in the current study are in line with those observed during healthy pregnancy (Jayasena et al., 2014c), as well as stimulating physiological processes such as oocyte maturation (Jayasena et al., 2014a) and restoring luteinizing hormone pulsatility (Jayasena et al., 2014b). Therefore, whilst our protocol does not precisely match normal physiology, we administered kisspeptin doses which have previously been shown to have physiological and sustained reproductive and behavioural effects.

In this study we selected the ACC as the region of interest as we have previously established that kisspeptin administration can enhance its activity in healthy men exposed to sexual and couple-bonding stimuli (Comninos et al., 2017). Furthermore, ACC GABA levels are known to closely relate to functional brain activity (Cleve et al., 2015, Northoff et al., 2007, Silveri et al., 2013). Due to the limited scanning-time available before potentially confounding downstream testosterone changes following kisspeptin infusion, as well as the prolonged time required to acquire robust spectroscopy signal directly from a single targeted region, further separate scanning studies focusing on other brain regions and other key neurotransmitters would be of particular interest. Indeed, these findings unlock a significant avenue for further research, to identify whether the effects of kisspeptin on GABA (or other neurotransmitters) in the ACC are also present in other key human brain regions (including those devoid of kisspeptin signalling).

We took several precautions to ensure the accuracy of the data and reduce potential confounders. Study visits were performed in a randomised order, with participants blinded as to the contents of the infusions. Crucially, each participant acted as their own control in a cross-over design to minimise inter-participant variability and enhance power. Observing the GABA signal within-participant over a short time period also lowered the likelihood that detected changes were driven by macromolecular differences; a known limitation of the MEGA-PRESS technique (Mullins et al., 2014). Additionally, all studies commenced in the morning to minimise the effect of circadian variation in reproductive hormone levels and MRS was completed prior to any increases in downstream testosterone.

In summary, we provide the first in vivo evidence for a kisspeptin-activated GABA pathway in humans that may underpin the central limbic brain effects of kisspeptin on the human brain. This initial study opens the door for further human work to identify the effects of kisspeptin on other brain regions and neurotransmitters using refinements of this technique. This has important implications for the current escalating development of kisspeptin-based therapies for related common reproductive disorders of body and mind.

## Declaration of Competing Interest

The authors declare the following financial interests/personal relationships which may be considered as potential competing interests: AA and WSD have undertaken consultancy for Myovant Sciences Ltd. WSD has undertaken consultancy for KaNDy Therapeutics.

## Footnotes

Appendix A Supplementary data associated with this article can be found in the online version at doi:10.1016/j.psyneuen.2021.105244.
