# Supplementary material for: Kisspeptin modulates gamma-aminobutyric acid levels in the human brain
Source: Psychoneuroendocrinology. 2021 Jul;129:105244. doi: 10.1016/j.psyneuen.2021.105244 (PMC8243259; doi:10.1016/j.psyneuen.2021.105244)
Supplement: Supplementary file 1 — Supplementary material [file mmc1.docx]

**MRS acquisition parameters**

MRS data were acquired using a MEGA-PRESS sequence (CMRR Spectroscopy C2P R2017-07, University Of Minnesota) with TR/TE =2000ms/68ms. Collection of 320 averages (160 edit-on and 160 edit-off acquisitions), resulted in a scan duration of 11 minutes. Water suppression was achieved using the VAPOR method at a bandwidth of 135Hz. During edit-on acquisitions, a frequency selective pulse (Bandwidth=55Hz) was applied at the GABA resonance at 1.9 ppm. For edit-off, the same pulse was applied at 7.46 ppm. Spectra were sampled with a bandwidth of 2000Hz and 2048 data points. An additional unsuppressed spectrum was acquired with 4 averages for water quantification.


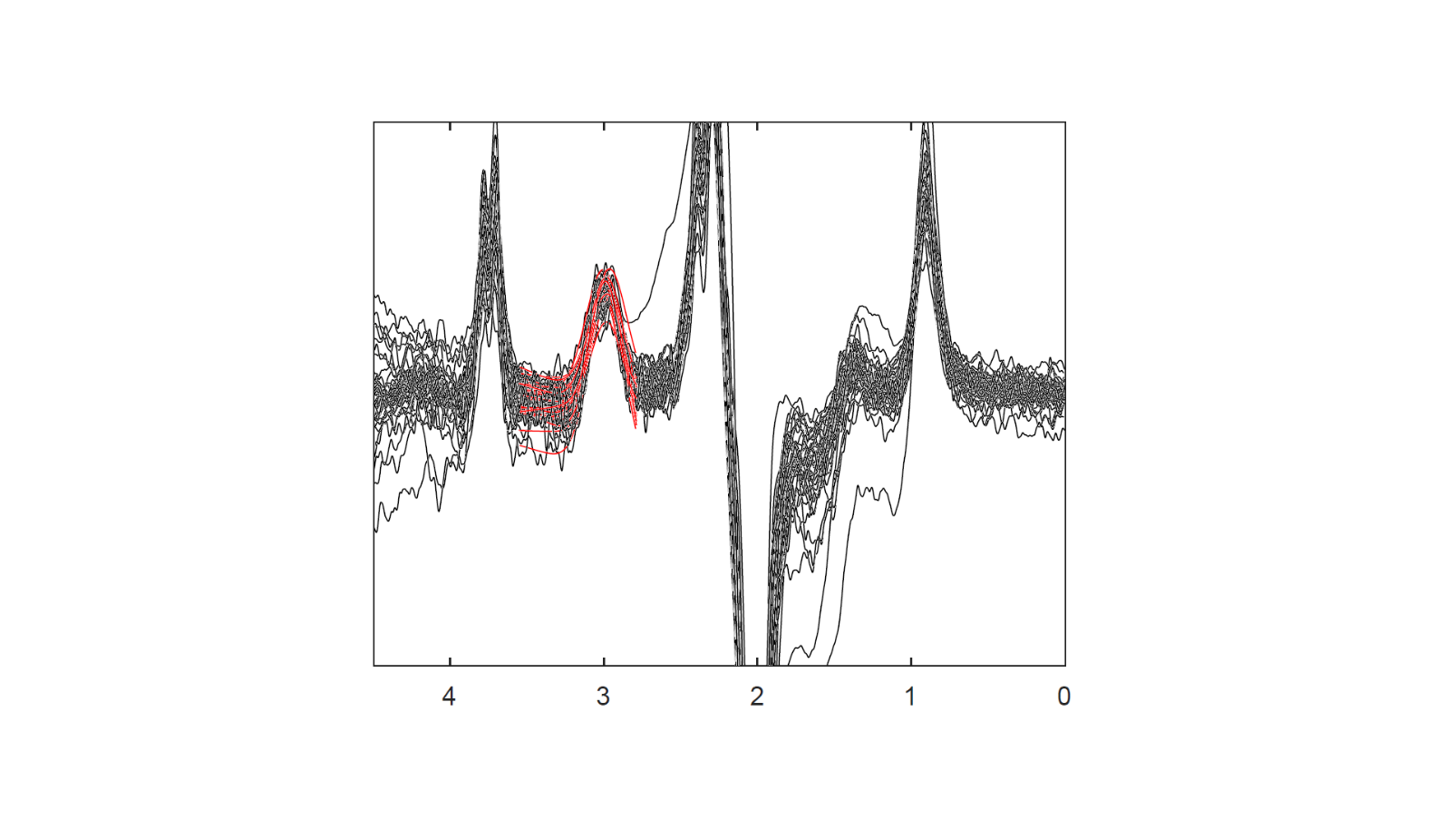
**Fitted spectra**

Frequency (ppm)

**Figure S1.** MRS spectra (black) overlaid for n=19 participants that met the 12% fitting criteria for both visits. Fitted GABA+ signal for each spectrum is shown in red.
